# Dietary Omega-3 Source Effect on the Fatty Acid Profile of Intramuscular and Perimuscular Fat—Preliminary Study on a Rat Model

**DOI:** 10.3390/nu12113382

**Published:** 2020-11-04

**Authors:** Katarzyna Czyż, Ewa Sokoła-Wysoczańska, Robert Bodkowski, Paulina Cholewińska, Anna Wyrostek

**Affiliations:** 1 Institute of Animal Breeding, Wrocław University of Environmental and Life Sciences, Chełmońskiego 38c, 51-630 Wrocław, Poland; robert.bodkowski@upwr.edu.pl (R.B.); paulina.cholewinska@upwr.edu.pl (P.C.); anna.wyrostek@upwr.edu.pl (A.W.); 2 The Lumina Cordis Foundation, Szymanowskiego 2a, 51-609 Wrocław, Poland; sokola@libero.it

**Keywords:** alpha-linolenic acid, eicosapentaenoic acid, docosahexaenoic acid, linseed oil, ethyl esters, fish oil

## Abstract

Fatty acids from the omega-3 family are an important element of both human and animal diets. Their activity involves a range of functions for the functioning of a whole organism, and their presence in animal diets can be considered as a means for animal origin product enrichment for human benefit or as compounds profitable for an animal’s health status. The aim of this preliminary study was to compare the effect of supplements rich in omega-3 fatty acids (linseed oil, linseed oil ethyl esters, and fish oil) in rat feed on the fatty acid profile of their intramuscular and perimuscular fat. The results demonstrated beneficial changes in fatty acid profiles (a decrease in saturated acids, an increase in unsaturated ones, i.e., omega-3 acids share) of examined tissues in the case of all supplements however, particular attention should be paid to linseed oil ethyl esters, which significantly increased the content of all omega-3 acids. Supplementation of animal diet with linseed oil ethyl esters may be beneficial for both animals, as omega-3 fatty acids exhibit profitable properties related to an animal’s health status and productivity, and humans who consume such enriched products.

## 1. Introduction

Alpha-linolenic acid (ALA) from the omega-3 family, commonly found in various plant sources, has long been the basic component of consumed fats, except for linoleic acid (LA) from the omega-6 group and oleic acid (OA) from the omega-9 family, in the so-called Paleolithic diet [1]. The role of this acid should not be underestimated as it is a precursor, and with the exception of fish oil and marine algae, is a source of other acids from the omega-3 family, i.e., eicosapentaenoic acid (EPA) and docosahexaenoic acid (DHA) for both humans and animals.

The acids from the omega-3 family are the basic lipid components of cell membranes and they are the substrates for the synthesis of regulatory substances in an organism. They participate in a range of important metabolic pathways, especially in the regulation and quenching of the inflammation state, and they are also a reservoir of energy. Unfortunately, due to the rapid development of agriculture in the 20th century, transition to industrial fodders in animal feeding, and also unprofitable changes in dietary habits of humans, these have almost totally disappeared as an element of a healthy diet. This has led to disturbances in the proper ratio of omega-6 to omega-3 acids in the diet of both humans and animals, which according to scientific reports should be 4.5:1 to 10:1, while currently in the Western diet the ratio is 15–16:1 or even 20:1 [2,3,4,5]. This is considered to be the main reason for the development of many chronic diseases observed in contemporary societies, including the so-called diseases of civilization, which include a variety of cancers, degenerative diseases, metabolic diseases, cardiovascular problems, diabetes, obesity, atherosclerosis, arteriosclerosis, ischemic heart diseases, immune system problems, allergies, dermatological diseases, gastrointestinal problems, hormonal problems, mental problems, memory disorders, Alzheimer’s disease, Parkinson’s, and emotional problems such as depression, aggression, and hyperactivity in children (ADHD) [6,7,8,9].

In recent years, research has led to changes in the understanding of chronic diseases. This concerns the nature of inflammation, i.e., the chronic low-grade systemic inflammation that has turned out to be the factor that over a period of many years induces most chronic diseases [10,11,12]. Increasing the amount of ALA in the diet is aimed at reducing the activity of omega-6 acids showing pro-inflammatory properties and increasing the amount of EPA and DHA acids with anti-inflammatory properties [13,14,15]. One of the most abundant sources of alpha-linolenic acid is linseed (also called flaxseed) (*Linum usitatissimum* L.) and linseed oil, which for this reason has attracted much attention as a potential dietary supplement both in human and animal nutrition [16,17,18]. However, with the exception of the beneficial fatty acid profile, linseed and linseed oil also contain some anti-nutrients like linatine, cyanogenic compounds, or phytic acid [16], which may raise some concern among consumers and to a some degree limit its dietary application. With regards to fish oil supplementation as a source of EPA and DHA fatty acids, it is believed that this can also be related to some risk resulting from the presence of environmental toxins, e.g., mercury, dioxins, polychlorinated biphenyls, or hypervitaminosis related to high levels of fat-soluble vitamin D and A in fish oil [19,20,21]. Therefore, the search for other supplementation possibilities seems to be justified.

The aim of our preliminary study was to compare the effect of different supplements containing omega-3 fatty acids of a plant and animal origin applied in Wistar rat feed on the profile of fatty acids in their intramuscular and perimuscular adipose tissue, with special attention paid to the level of ALA, EPA, and DHA acids. The rationale for the study was an attempt to determine the profile of fatty acids in intramuscular and perimuscular fat on a rat model, with the possible use of tested supplements in the feeding of farm animals as a source of products that are an important part of the human diet.

## 2. Materials and Methods

Ethyl esters of linseed oil rich in alpha-linolenic acid were synthesized according to the technology elaborated at the University of Wrocław (Poland) [22]. The technology of production and characteristics of linseed oil ethyl ester applied in this study are presented in Sokoła-Wysoczańska et al. [23]. Briefly, the technology is based on oil transesterification (a mixture of triglycerides of higher omega-3, -6, -9 fatty acids) with ethanol in the presence of a catalyst. The process consists of several stages. The first one is transesterification in an anaerobic atmosphere, then the removal of unreacted bioethanol from the post-reaction mixture, followed by a separation of the glycerin phase from the raw ester phase in gravity separators. In the next stage, the raw esters are cleaned by centrifugation and then cleaned by means of a residual gas alcohol depot with nitrogen and by sedimentation of the residual glycerin phase. Finally, the glycerin phase is separated.

Raw linseed oil, which was a substrate for the production of ethyl esters, and commercially available fish oil (cod liver) were also used in the study for comparative purposes. The content of selected fatty acids in supplements is provided in Table 1.

The study was conducted on Wistar rats from the monozygotic Charles Rivers Laboratories (Germany). Male animals with an initial body weight of about 380 g were kept individually in cages in the vivarium of the Faculty of Veterinary Medicine, Wrocław University of Environmental and Life Sciences, Poland, at a temperature of about 21 °C with a 12 h light/dark cycle. The animals were fed with standard laboratory animal feed (diet no. C 1090-10) from Altromin International (Germany) and had ad libitum access to water. Before the experiment, the rats were subjected to an acclimatization period of 6 weeks. The experiment lasted 8 weeks, during which time the rats from the experimental groups were additionally supplemented with linseed oil (the raw material for the production of ethyl esters), linseed oil ethyl esters, and fish oil. All supplements were administered orally using a syringe: 0.04 g/kg body weight.

The animals were randomly divided into the following groups: Group C—control, group LO—receiving linseed oil, group EE—receiving linseed oil ethyl esters, and group FO—receiving fish oil. Each group consisted of 8 animals. The animal’s body weight was recorded on the day of the beginning of the experiment and on the last day, and the body weight gains were calculated. On the last day of the experiment, all animals were euthanized and samples of intramuscular and perimuscular fat were collected to determine the fatty acid profile of the collected material. The study was carried out with the agreement of the 2nd Local Animal Ethics Committee, Wrocław University of Environmental and Life Sciences, Poland (approval no. 79/2010).

Fatty acid profile in the collected material was determined according to the method described by Kroger et al. [24], while fatty acid methyl esters (FAME) prepared employing the method developed by Prescha et al. [25]. An analysis of FAME was performed with a 6890 N gas chromatograph (Agilent Technologies, Santa Clara, CA, USA) equipped with a flame ionization detector (FID) and an Rtx 2330 100 m × 0.25 mm × 0.5 um capillary column (Restek, Bellefonte, PA, USA). Hydrogen was used as the carrier gas at a flow rate of 1.5 mL/min and the separation was carried out at a temperature set from 165 °C (for 10 min) to 220 °C, the temperature being increased at a rate of 2 °C/min. The identification of individual fatty acids was accomplished through a comparison with external standards. Pentadecanoic acid was used as an internal standard for quantitative analysis and Chemstation vB.04.02 (Agilent Technologies, Santa Clara, CA, USA) was used to calculate the results.

Data on fatty acids are presented as a percentage of individual acids in the total acid pool. Total share of saturated acids (SFA), unsaturated fatty acids (UFA), monounsaturated fatty acids (MUFA), and polyunsaturated fatty acids (PUFA), as well as their ratios were calculated. In addition, the content of total n-3, n-6, and n-9 fatty acids was determined.

The lipid quality indices, i.e., atherogenic index (AI) and thrombogenic index (TI), were calculated based on the fatty acid profiles of examined samples according to the following formulas [26,27]:

AI = (C12:0 + 4 × C14:0 + C16:0)/(n-6 PUFA + n-3PUFA + MUFA) (1)

TI = (C14:0 + C16:0 + C18:0)/(0.5 × n-6PUFA + 3 × n-3PUFA + n-3PUFA/n-6PUFA)(2)

The results were analyzed statistically using Statistica 13.0 (StatSoft, Krakow, Poland) and presented as mean values and standard deviation (SD). The normality of distribution of the obtained results was checked using the Shapiro–Wilk test, followed by the one-factor ANOVA. The significance of differences between the groups was determined using Tukey’s test at the significance level of *p* < 0.05.

## 3. Results

Table 2 presents the results concerning the body weight of the examined rats. No statistically significant differences were found in the body weight of the animals both before and after the experiment, and in addition their body weight gains were at a similar level. It is also worth noting that no disease symptoms like e.g., diarrhea were found during the whole period of the study.

**Figure 1:**
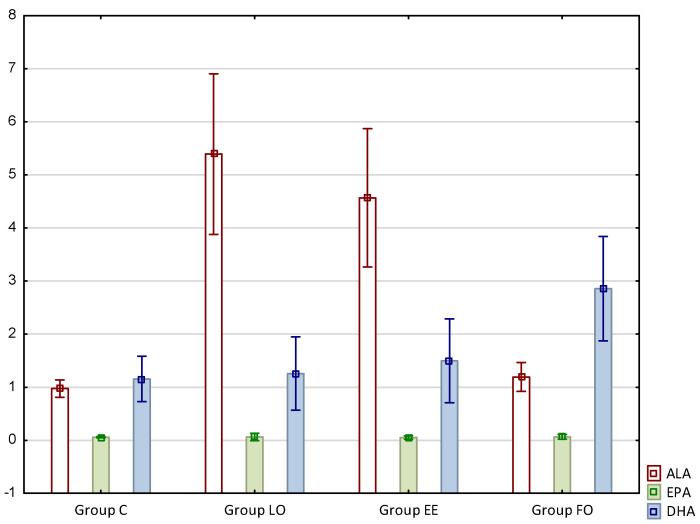
The content of omega-3 acids in rat intramuscular fat (% of total fatty acids). ALA- alpha-linolenic acid; EPA—eicosapentaenoic acid; DHA—docosahexaenoic acid.

The results concerning fatty acid profiles of the intramuscular fat of rats are presented in Table 3 and Table 4 as well as in Figure 1.

Among the saturated fatty acids, the highest content in all groups was noted for palmitic acid (C16:0). Its level was significantly reduced (by about 7%) in the LO group compared to the control. A statistically significant difference was also confirmed between the content of this acid in the LO and FO groups, in which its content was over 6% higher than in the LO group (Table 3). Other differences that turned out to be statistically significant were noted in the case of myristic acid (C14:0) between groups LO and EE and group FO, where the levels were 16% and 13% higher than those for LO and EE, respectively. The content of lignoceric acid (C24:0), despite being generally low in all the groups, increased significantly in the experimental groups compared to the control.

With regard to the total pool of saturated fatty acids (SFA) (Table 4), statistically significant differences were noted between groups LO and FO (about 6% higher level in the FO group).

The composition of intramuscular unsaturated fatty acids was more diversified (Table 3). The acids that were found in the highest amounts were oleic acid (C18:1n9c) and linoleic acid (C18:2n6c), and then palmitoleic acid (C16:1). Statistically significant differences were noted between linoleic acid (LA) content in group C and groups LO and EE, or between the content of this acid in group FO and the groups receiving supplements based on linseed oil (i.e., LO and EE). The increase in LA acid content in these groups compared to the control was about 18% and 20%, respectively. In turn, palmitoleic acid content decreased nearly two-fold in groups LO and EE with respect to the control, and the differences were significant. Another acid that content decreased significantly in groups LO and EE was margaroleic acid (C17:1), while the content of arachidonic acid (C20:4n6) was significantly lower (by about 46%) in group FO compared to the control. An over four-fold increase in the level of 11-eicosenoic acid (C20:1n9) in group FO compared to the control should also be noted.

Taking into account fatty acids from the omega-3 series, it is worth emphasizing that the content of alpha-linolenic acid (C18:3n3, ALA) increased significantly in the groups supplemented with linseed oil or ethyl esters based thereon, i.e., LO and EE, and this increase was about 5.5-fold and 4.6-fold, respectively. In turn, the content of docosahexaenoic acid (C22:6n3, DHA) was the highest in the group supplemented with fish oil (FO), and this was about 145% higher compared to the control, and 90% higher compared to group EE (Table 3, Figure 1).

Interestingly, when considering the total pool of unsaturated fatty acids (UFA), a significantly confirmed difference can only be found between groups LO and FO. Total monounsaturated fatty acid content (MUFA) decreased significantly in groups LO and EE compared to the control (by about 10% and 11%, respectively), while the content of polyunsaturated fatty acids (PUFA) increased in groups LO and EE (by about 29% and 26%, respectively), and in these groups it was also significantly higher when compared to group FO (by about 23% and 21%, respectively).

A statistically confirmed increase was observed in the case of PUFA/MUFA as well as PUFA/UFA acid ratios in groups LO and EE compared to the control. In the former, the increase was about 44% for both groups (LO and EE), while in the case of the PUFA/UFA ratio, the value increased by about 26% for both groups. The UFA/SFA ratio differed significantly between groups LO and FO.

Other indices examined in the study concerned the contents of total n-3, n-6, and n-9 acids, as well as the n-6/n-3 ratio (Table 4). In the case of n-3 acids, a statistically significant increase was noted for all experimental groups compared to the control however, the highest increase was observed for group LO, then for EE (about 3-and 2.8-fold, respectively). The content of n-6 fatty acids in group LO increased by about 10%, while in the case of group EE it was 12%. As a consequence, the ratio of n-6/n-3 acids decreased in all experimental groups compared to the control. The lowest value of this ratio was found for group LO, where it was about 2.8-fold lower compared to the control, while for groups EE and FO this value was about 2.5-and 1.9-fold lower, respectively.

The values of atherogenic (AI) and thrombogenic (TI) indices calculated based on the results of fatty acid analysis for intramuscular fat are presented in Table 4. Significant differences in the case of both indices were noted between the control and groups LO and EE, and this decrease amounted to about 10% and 7% in the case of AI, or 28% and 23% for TI, respectively. In addition, the differences between groups FO and LO or EE were confirmed statistically in both cases.

**Figure 2:**
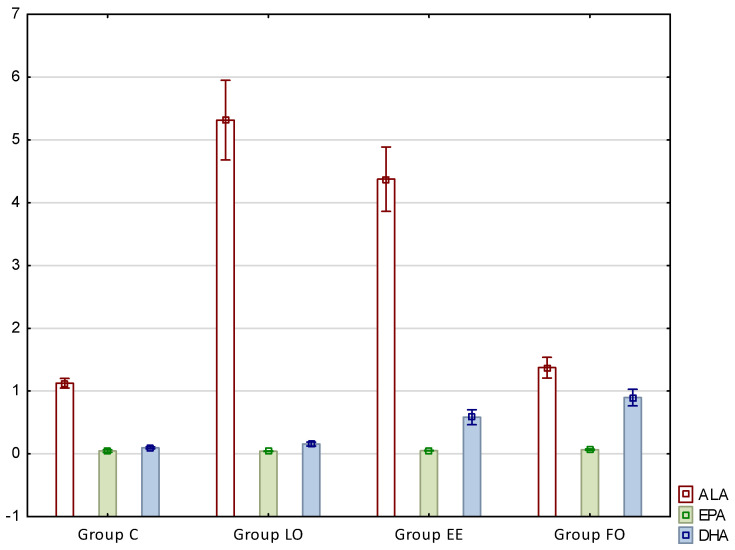
The content of omega-3 acids in rat intramuscular fat (% of total fatty acids). ALA—alpha-linolenic acid; EPA—eicosapentaenoic acid; DHA—docosahexaenoic acid.

The results presenting fatty acid profiles of perimuscular fat of rats are presented in Table 5 and Table 6 as well as in Figure 2.

As in the case of intramuscular fat, the highest content among the saturated fatty acids in all groups was noted for palmitic acid (C16:0). The content of this acid decreased significantly in groups LO and EE compared to the control (by about 8% and 7%, respectively). In the case of the FO group, the content of this acid was significantly higher compared to groups LO and EE. In addition, statistically significant differences were noted for stearic acid (C18:0) between the control and experimental groups however, in this case an increase was observed. Other significant differences were found in the case of myristic acid (C14:0) however, only between groups LO and FO, and for margaric acid (C17:0) between the control and all experimental groups, as well as in the case of arachidic acid (C20:0) between the control and group FO, and for lignoceric acid (C24:0) between the control and LO groups and FO.

In the case of total pool of saturated fatty acids, the differences turned out to be statistically significant between groups LO and EE and FO (Table 6).

Among the unsaturated fatty acids, the highest amounts were noted for oleic acid (C18:1n9c) and linoleic acid (C18:2n6c), and then palmitoleic acid (C16:1), as in the case of intramuscular fat. In the case of palmitoleic acid a significant decrease was found between the control and groups LO and EE (about 2.1-and 1.6-fold, respectively). A statistically confirmed reduction compared to the control group was also observed for margaroleic acid (C17:1) in the case of groups LO and EE, eicosadienoic acid (C20:2) for LO group, and arachidonic acid (C20:4n6) for groups LO and EE. In addition, the content of gamma-linolenic acid from the omega-6 series was significantly reduced in group LO compared to the control. The content of 11-eicosenoic acid (C20:1n9) increased significantly, about 4.3-fold, in group FO compared to the control.

As in the case of intramuscular fat, beneficial results were noted with regards to omega-3 fatty acid profiles, i.e., ALA, and DHA. The content of alpha-linolenic acid increased significantly in groups LO and EE, and this increase was about 4.7- and 3.8-fold, respectively. The values in these groups were also significantly higher compared to group FO. In the case of docosahexaenoic acid content, an increase was observed between the control and LO and groups EE and FO (about 4.5- and 7-fold, respectively), and concurrently the difference between groups EE and FO was also significant (Table 4, Figure 2). In the case of eicosapentaenoic acid (C20:5n3, EPA), a significant difference was noted between group FO and the other ones.

Statistically confirmed differences in the total pool of unsaturated fatty acids were found between groups LO or EE and FO. While the content of monounsaturated fatty acids was significantly reduced in groups LO and EE compared to the control (by about 7.5% and 8%, respectively), the content of polyunsaturated fatty acids increased significantly in groups LO and EE compared to the control, and the observed increase was about 36% and 25%, respectively. In the case of group FO, statistically confirmed differences were noted with respect to groups LO and EE.

Table 6 also presents values concerning total n-3, n-6, and n-9 acid content. Differences confirmed statistically were observed in the case of n-3 acid content between the control and groups LO and EE, and increases were about 4.3-and 3.9-fold, respectively. In turn, with regards to n-6 fatty acid content, a statistically significant difference was only noted between groups EE and FO, and the same situation was found for the n-9 fatty acid content. However, the ratio of n-6/n-3 was significantly reduced in all experimental groups and the most profitable value with respect to the control was observed for group LO, then EE, and this difference was the smallest, however still significant, in the case of group FO (about 4-, 3.6-, and 1.9-fold decrease, respectively).

The values of atherogenic (AI) and thrombogenic (TI) indices for perimuscular fat are presented in Table 6. As in the case of intramuscular fat, significant differences in the case of both indices were noted between the control and groups LO and EE. The reduction was about 10% and 7% in the case of AI, or 28% and 24% for TI, respectively. In addition, the differences between groups FO and LO or EE were confirmed statistically in both cases.

With regards to the ratios of particular groups of fatty acids, i.e., SFA, UFA, MUFA, and PUFA, a statistically significant increase was found for PUFA/MUFA ratios between the control and groups LO and EE, which was also observed in the case of PUFA/UFA. These indices were also significantly different for these groups and group FO. The UFA/SFA ratio was significantly different between groups LO or EE and FO.

## 4. Discussion

The results of this study can be evaluated in two ways. The first relates to animal origin food enrichment with omega-3 fatty acids, which can lead to the production of so-called functional food and thus beneficial health-promoting effects in the consumers of such food. The second aspect is the effect of dietary supplementation of animals with omega-3 fatty acid sources on their health status and productivity, thus generating benefits for the animal organism and concurrent production from such supplementation.

With regard to the first aspect, issues related to animal origin food biofortification or enrichment with biologically active substances, including fatty acids, have been widely studied and reported in the literature. However, there is an ongoing search for the most effective forms of supplements possible; i.e., ones that would be maximally utilized by animal organisms and be transferred to products such as meat, milk, or eggs. One of the most important components of the human diet is meat as it provides proteins and fats, as well as many essential microelements and vitamins [28]. However, very often negative effects of consumption of animal-derived fats overshadow possible advantages, which to a large degree results from the relatively high content of saturated fatty acids (SFAs), especially those with thrombogenic and atherogenic activity, e.g., myristic or palmitic ones [29,30]. The results obtained in our study indicate beneficial changes in both analyzed tissues as a result of supplementation with linseed oil and linseed oil ethyl esters.

In general, there are several factors that can influence meat FA profiles and these include breed, genotype, gender, environmental conditions, diet, and also muscle type [31,32]. Obviously, there are some significant differences between groups of animals with respect to their ability to absorb and transform fatty acids derived from the diet. In the case of non-ruminant animals, the FA profile of their diets is directly reflected in their tissues and thus products obtained from these animals [31]. In turn, ruminants are characterized by a low content of polyunsaturated fatty acids (PUFA) which is due to microbial biohydrogenation in the rumen, which causes a limited outflow of FAs in rumen and further absorption in the intestines [33]. The unique feature of this group of animals is the preferential incorporation of long chain omega-3 fatty acids into phospholipids and not into triacylglycerol [34]. Meat from sheep mainly contains medium-to long-chain FAs, and compared to beef meat, it is characterized by a higher share of omega-3 and omega-6 PUFAs. This is related to the fact that the rumen of sheep is smaller compared to that of cattle, and therefore biohydrogenation that occurs in rumen is completed to a lower degree. Retention times are shorter in sheep and rates of feed passage from the rumen are higher, which subsequently enhances duodenal flow, as well as the absorption and deposition of FAs in tissues [35].

There are various strategies aimed at improving meat lipid profiles and many of these include an increase in long-chain polyunsaturated fatty acid (LC-PUFA) content, especially those from the omega-3 family, i.e., alpha-linolenic acid (ALA, 18:3, n-3), eicosapentaenoic acid (EPA, 20:5, n-3), and docosahexaenoic acid (DHA, 22:6, n-3), and the supplements used in this study resulted in such profitable changes in the FA profiles of the analyzed samples. While ALA can be found in plant sources (e.g., linseed or linseed oil), the sources of EPA and DHA are of marine origin, i.e., fish oil or algae [36,37]. It has been demonstrated that fish oil is characterized by a higher rate of ruminal biohydrogenation compared to marine algae, thus the latter is more effective in omega-3 LC-PUFAs level increases in animal tissues [34]. In the case of plant sources, such as linseed or linseed oil, it must be remembered that although a diet rich in ALA leads to a noticeable increase in omega-3 PUFA level, the degree of ALA ruminal biohydrogenation is high, usually in the range of 90% [38].

Another problem that should be taken into account is competition between linoleic acid (LA, 18:2, n-6) and ALA for the same pathways in LC-PUFA synthesis and thus for the availability of the same enzymes, i.e., elongases and desaturases, especially Δ6-desaturase. Therefore, an excessive intake of LA may cause the level of this enzyme available for ALA metabolism to be insufficient, and thus the conversion of ALA to EPA and DHA may be limited [8]. With regard to ruminant animals, it is suggested that LA has lower rates of biohydrogenation in rumen compared to ALA [38,39], and thus it can be more easily converted into arachidonic acid (AA, 20:4, n-6) and incorporated into various tissues. For this reason, a diet with high levels of ALA seems to be a good solution as it may overcome problems related to competition between LA and ALA resulting in a considerably higher rate of ALA conversion to EPA and DHA, which is beneficial for both human and animal organisms. For this reason, linseed oil-based supplements may be a good choice, which was also confirmed in this study. For example, in our previous study [40] we demonstrated that the application of linseed oil ethyl esters in the diet of dairy cows and sheep beneficially modified the profile of FAs in their milk, including a significant decrease in saturated fatty acid content and a concurrent increase in the content of unsaturated acids with about 22% in sheep milk and 20% in case of cows. The content of alpha-linolenic acid increased by 42% in sheep and 59% in cow milk.

The other aspect mentioned above is the effect of animals’ dietary supplementation with omega-3 fatty acids on their health status and productivity. It was demonstrated for example in the study conducted by Candynine et al. [41] that linseed oil addition to the diet of sheep and goats resulted in an improved feed conversion rate (FCR) in these animals, and the authors explain this on the basis of the caloric value of the oil, and thus higher energy intake by animals as a result of supplementation. Consequently, average daily gains were also subject to an increase (by about 30%) which can be an attractive for meat producers. Additionally, no deterioration in nutrient digestibility as a result of linseed oil introduction to the diet was observed, and in some cases it was even improved [41].

Many studies have demonstrated an important role of polyunsaturated fatty acids in reproduction, which is one of the conditions of animal productivity [42,43,44]. It is also important with regard to issues related to semen storage and its freezing-thawing before use in artificial insemination (AI) which is a common practice in the case of some animal species. Mammalian sperm is composed of two main elements, i.e., head and tail, and each of these plays an important role in the series of processes related to fertilization [42]. The functions of the sperm head are mostly associated with acrosome reaction and membrane fusion, while the tail is mostly responsible for sperm movement [45], thus lipid distribution in these sperm elements is uneven [46]. It has been demonstrated that the vast majority of DHA is located in the sperm tail and an increase in this acid content, and generally the content of omega-3 fatty acids in sperm leads to an increased fluidity and flexibility of the sperm membrane and flagellum, which in turn prevents a decrease in motility parameters during cryopreservation [47,48]. Thus, high quality sperm is related to its high content of omega-3 FAs, in particular DHA, and its dietary omega-6 to omega-3 ratio, the reduction of which has also been associated with benefits for both fertility and general health status [49,50]. The freezing-thawing procedure may have a negative effect on the integrity of the sperm membrane and can cause a decrease in the level of PUFAs in semen used for AI [51]. The study conducted on bulls by Khoshniat et al. [42] demonstrated that linseed oil supplementation of animal diet resulted in increased sperm survival, sperm membrane integrity in thawed semen, and also a marked decrease in the percentage of abnormal spermatozoa. The sperm of linseed oil-supplemented animals also demonstrated significant improvement in total and progressive motility as well as other sperm motion features after the freezing-thawing procedure [42].

Linseed oil ethyl esters applied in the study are characterized by a few- to several-fold higher bioavailability compared to the traditional triglyceride form, and are better absorbed and incorporated into various lipid fractions of blood [52], which is mainly caused by their simple molecular structure and more efficient kinetics of free acid release, ensuring more efficient digestion. Ethyl esters of linseed oil are characterized by very low toxicity, comparable to natural plant triglycerides, and an absence of very toxic substances from the cyanohydrate group (amygdalin, linamarin) present in the oil, which strongly damages liver cells, and complete resistance to the processes of acidic hydrolysis in liver environment which provides the possibility of transport to further parts of a gastrointestinal tract. Concurrently, they contain some health-promoting components of linseed oil such as lignans, phytoestrogens, and other phytosterols (campesterol, sitosterol), carotenoids (β-carotene), phospholipids, and other natural components. Oxygen solubility in esters is considerably lower compared to oil, which is reflected in a considerable increase in stability over longer time periods. They are also significantly less susceptible to the processes of oxidation, epoxidation, and peroxidation than is the case with the raw material they are produced from (linseed oil) [23,40].

Moreover, in our study we observed a higher conversion rate of ALA to EPA and DHA for linseed oil ethyl esters compared to the oil than that reported in the literature. The esters are produced in a nitrogen atmosphere without oxygen access therefore, we assume that the most important factor that may reduce the conversion of alpha-linolenic acid is related to the products of alpha-linolenic acid auto-oxidation. Thus, it can be suggested that the preserved double bonds and lack of auto-oxidation products are the two main factors that guarantee a successful conversion of alpha-linolenic acid. However, this requires further studies entirely focused on the conversion rate and the lack of such an analysis is undoubtedly one of the limitations of the study presented. Other limitations include a low number of animals in particular groups, as well as a lack of results concerning molecular mechanisms underlying the observed changes.

## 5. Conclusions

The study demonstrated that linseed oil ethyl esters may be a valuable source of omega-3 fatty acids, i.e., alpha-linolenic acid and its metabolites EPA and DHA, in animal diets supplementation aimed at animal origin products enrichment in pro-healthy fatty acids. Such supplementation of animals may be beneficial for both animals, as omega-3 fatty acids exhibit profitable properties related to animals health status and productivity, and humans who consume such enriched products.

## Figures and Tables

**Table 1:** Fatty acid profiles of supplements used in the study (% of total fatty acids).

Acid	LO	EE	FO
C16:0	4.37	4.44	11.36
C18:0	3.79	3.43	2.68
C18:1	16.41	16.73	23.95
C18:2	16.24	16.68	1.43
C18:3	56.29	58.71	−
C20:5	−	−	8.13
C22:6	−	−	9.87

LO—linseed oil group; EE—linseed oil ethyl esters group; FO—fish oil group.

**Table 2:** Body weight at the beginning and the end of the experiment (g).

Parameter	Group C		Group LO		Group EE		Group FO	
	Mean	SD	Mean	SD	Mean	SD	Mean	SD
Initial body weight	376.94	12.10	382.45	19.05	379.51	12.60	384.30	14.25
Final body weight	557.16	24.68	578.71	15.49	569.38	26.40	593.64	30.13
Body weight gains	180.23	24.00	196.23	16.41	189.87	33.27	209.34	42.27

SD—standard deviation.

**Table 3:** Fatty acid profile in rat intramuscular fat (% of total fatty acids).

Fatty Acid	Group C		Group LO		Group EE		Group FO	
	Mean	SD	Mean	SD	Mean	SD	Mean	SD
Saturated fatty acids								
C12:0	0.48	0.03	0.55	0.06	0.54	0.10	0.62	0.23
C14:0	2.98	0.05	2.71 ^a^	0.25	2.80 ^a^	0.14	3.22 ^b^	0.31
C16:0	26.50 ^a^	0.12	24.55 ^b^	0.76	25.32	0.93	26.13 ^a,c^	0.60
C17:0	0.31	0.02	0.35	0.05	0.35	0.04	0.34	0.01
C18:0	4.17	0.82	5.01	1.09	5.05	0.78	4.62	0.77
C20:0	0.07	0.01	0.07	0.03	0.07	0.01	0.07	0.02
C24:0	0.08 ^a^	0.01	0.23 ^b^	0.04	0.22 ^b^	0.05	0.51 ^c^	0.10
Unsaturated fatty acids								
C16:1	8.73 ^a^	1.36	4.24 ^b^	0.60	4.45 ^b^	1.09	5.95	0.63
C17:1	0.35 ^a^	0.01	0.28 ^b^	0.04	0.29 ^b^	0.02	0.34 ^a,c^	0.02
C18:1n9c	35.81	0.68	35.75	1.71	35.06	1.81	36.04	1.68
C18:2n6c	14.15 ^a^	0.28	16.70 ^b^	1.23	16.98 ^b^	0.96	14.74 ^a,c^	0.74
C18:3n6	0.06	0.01	0.06	0.03	0.06	0.02	0.05	0.01
C18:3n3	0.98 ^a^	0.08	5.39 ^b^	0.76	4.57 ^b,d^	0.65	1.19 ^a,c,d^	0.14
C20:1n9	0.25 ^a^	0.05	0.26 ^a^	0.03	0.26 ^a^	0.02	1.08 ^b^	0.17
C20:2	0.12	0.01	0.13	0.02	0.13	0.01	0.16	0.03
C20:4n6	2.53 ^a^	0.61	1.68	0.63	1.69	0.45	1.36 ^b^	0.29
C20:5n3	0.06	0.01	0.06	0.04	0.05	0.02	0.07	0.02
C22:6n3	1.16 ^a^	0.21	1.26 ^a^	0.35	1.50 ^b^	0.40	2.85 ^c^	0.49
other	1.21 ^a^	0.48	0.85 ^b^	0.78	0.61 ^b^	0.06	0.65 ^b^	0.15

^a–d^ Different superscripts indicate statistical differences between the groups at *p* < 0.05.

**Table 4:** Summary of fatty acid content in rat intramuscular fat (% of total fatty acids).

	Group C		Group LO		Group EE		Group FO	
	Mean	SD	Mean	SD	Mean	SD	Mean	SD
Total SFA	34.60	0.90	33.46 ^a^	0.95	34.36	1.09	35.52 ^b^	1.52
Total UFA	65.40	0.90	66.67 ^a^	0.86	65.64	1.09	64.48 ^b^	1.52
Total MUFA	45.14 ^a^	1.82	40.54 ^b^	1.61	40.05 ^b^	2.48	43.41	1.93
Total PUFA	20.27 ^a^	1.16	26.13 ^b^	1.46	25.59 ^b^	1.67	21.07 ^a,c^	1.11
PUFA/MUFA	0.45 ^a^	0.04	0.65 ^b^	0.06	0.64 ^b^	0.08	0.49 ^a,c^	0.04
PUFA/UFA	0.31 ^a^	0.02	0.39 ^b^	0.02	0.39 ^b^	0.03	0.33 ^a,c^	0.02
UFA/SFA	1.89	0.09	2.00 ^a^	0.08	1.91	0.10	1.82 ^b^	0.13
Total n-3	2.19 ^a^	0.22	6.71 ^b^	0.64	6.12 ^b,d^	0.64	4.11 ^a,c,d^	0.55
Total n-6	16.74 ^a^	0.85	18.44 ^b^	1.50	18.74 ^b^	1.28	16.15 ^a,c^	0.70
Total n-9	36.06	0.65	36.02	1.71	35.32	1.82	37.12	1.71
n-6/n-3 ratio	7.69 ^a^	0.55	2.77 ^b^	0.36	3.09 ^b,d^	0.31	3.98 ^a,c,d^	0.50
AI	0.60 ^a^	0.01	0.54 ^b^	0.03	0.56 ^b,d^	0.02	0.61 ^a,c,d^	0.04
TI	0.90 ^a^	0.04	0.65 ^b^	0.03	0.69 ^b,d^	0.03	0.80 ^a,c,d^	0.04

^a–d^ Different superscripts indicate statistical differences between the groups at *p* < 0.05. Share of saturated acids (SFA), unsaturated fatty acids (UFA), monounsaturated fatty acids (MUFA), polyunsaturated fatty acids (PUFA), atherogenic index (AI), and thrombogenic index (TI).

**Table 5:** Fatty acid profiles in rat perimuscular fat (% of total fatty acids).

Fatty Acid	Group C		Group LO		Group EE		Group FO	
	Mean	SD	Mean	SD	Mean	SD	Mean	SD
Saturated fatty acids								
C12:0	0.48	0.03	0.51	0.05	0.56	0.05	0.55	0.07
C14:0	2.90	0.16	2.70 ^a^	0.25	2.95	0.18	3.17 ^b^	0.26
C16:0	27.01 ^a^	0.75	24.87 ^b^	1.00	25.25 ^b^	0.38	26.62 ^a,c^	0.57
C17:0	0.29 ^a^	0.02	0.35 ^b^	0.03	0.35 ^b^	0.01	0.36 ^b^	0.02
C18:0	3.42 ^a^	0.14	4.45 ^b^	0.48	4.05 ^b^	0.15	4.16 ^b^	0.38
C20:0	0.12 ^a^	0.05	0.06	0.01	0.07	0.02	0.06 ^b^	0.01
C24:0	0.08 ^a^	0.05	0.09 ^a^	0.01	0.15	0.09	0.28 ^b^	0.05
Unsaturated fatty acids								
C16:1	6.86 ^a^	0.59	3.23 ^b^	0.98	4.21 ^b,c^	0.36	4.77	0.44
C17:1	0.31 ^a^	0.01	0.24 ^b^	0.03	0.26 ^b^	0.01	0.28	0.01
C18:1n9c	39.57	0.25	39.73	0.92	38.38	0.94	39.63	1.00
C18:2n6c	16.15	0.85	17.18	1.44	17.35	0.79	15.71	1.05
C18:3n6	0.10 ^a^	0.05	0.04 ^b^	0.01	0.06	0.02	0.06	0.03
C18:3n3	1.13 ^a^	0.09	5.32 ^b^	0.76	4.38 ^b,d^	0.61	1.37 ^a,c,d^	0.20
C20:1n9	0.26 ^a^	0.02	0.26 ^a^	0.04	0.33 ^a^	0.13	1.12 ^b^	0.13
C20:2	0.14 ^a^	0.02	0.10 ^b^	0.02	0.12	0.01	0.14 ^a,c^	0.02
C20:4n6	0.33 ^a^	0.03	0.24 ^b^	0.03	0.26 ^b^	0.03	0.26	0.07
C20:5n3	0.05 ^a^	0.02	0.04 ^a^	0.01	0.05	0.01	0.07 ^b^	0.01
C22:6n3	0.10 ^a^	0.02	0.16 ^a,b^	0.04	0.58 ^c^	0.14	0.90 ^d^	0.16
other	0.72	0.39	0.45	0.09	0.64	0.21	0.51	0.15

^a–d^ Different superscripts indicate statistical differences between the groups at *p* < 0.05.

**Table 6:** Summary of fatty acid content in rat perimuscular fat (% of total fatty acids).

	Group C		Group LO		Group EE		Group FO	
	Mean	SD	Mean	SD	Mean	SD	Mean	SD
Total SFA	34.29	0.73	33.03 ^a^	1.36	33.38 ^a^	0.44	35.19 ^b^	0.99
Total UFA	65.71	0.73	66.97 ^a^	1.36	66.62 ^a^	0.44	64.81 ^b^	0.99
Total MUFA	47.00 ^a^	0.44	43.45 ^b^	1.21	43.18 ^b^	1.10	45.80	1.23
Total PUFA	18.71 ^a^	1.06	25.52 ^b^	1.72	23.44 ^b^	1.20	19.01 ^a,c^	1.14
PUFA/MUFA	0.40 ^a^	0.03	0.54 ^b^	0.05	0.54 ^b^	0.04	0.42 ^a,c^	0.03
PUFA/UFA	0.29 ^a^	0.01	0.35 ^b^	0.02	0.35 ^b^	0.02	0.29 ^a,c^	0.02
UFA/SFA	1.92	0.06	2.03 ^a^	0.12	2.00 ^a^	0.40	1.84 ^b^	0.08
Total n-3	1.27 ^a^	0.10	5.52 ^b^	0.79	5.01 ^b,d^	0.61	2.34 ^a,c,d^	0.33
Total n-6	16.58	0.91	17.46	1.44	17.67 ^a^	0.80	16.03 ^b^	1.05
Total n-9	39.83	0.25	39.99	0.93	38.71 ^a^	0.96	40.75 ^b^	1.00
n-6/n-3 ratio	13.13 ^a^	0.94	3.21 ^b^	0.48	3.56 ^b,d^	0.38	6.95 ^a,c,d^	0.87
AI	0.60 ^a,c,d^	0.02	0.54 ^b,d^	0.04	0.56 ^d^	0.02	0.61 ^c^	0.03
TI	0.94 ^a^	0.03	0.68 ^b^	0.06	0.71 ^b^	0.03	0.89 ^a,c^	0.05

^a–d^ Different superscripts indicate statistical differences between the groups at *p* < 0.05.
